# H1/pAIM2 nanoparticles exert anti‐tumour effects that is associated with the inflammasome activation in renal carcinoma

**DOI:** 10.1111/jcmm.13842

**Published:** 2018-08-30

**Authors:** Dafei Chai, Nianli Liu, Huizhong Li, Gang Wang, Jingyuan Song, Lin Fang, Zheng Lu, Hong Yao, Junnian Zheng

**Affiliations:** ^1^ Cancer Institute Xuzhou Medical University Xuzhou China; ^2^ Center of Clinical Oncology Affiliated Hospital of Xuzhou Medical University Xuzhou Medical University Xuzhou China; ^3^ Jiangsu Center for the Collaboration and Innovation of Cancer Biotherapy Cancer Institute Xuzhou Medical University Xuzhou China

**Keywords:** AIM2, H1 nanoparticles, inflammasome, renal cell carcinoma, treatment

## Abstract

Renal cell carcinoma (RCC) is a high metastasis tumour with less effective treatment available currently. Absent in melanoma 2 (AIM2) as a tumour suppressor might be used as a potential therapeutic target for RCC treatment. Here, we found that AIM2 expression was significantly decreased in RCC patient specimens and renal carcinoma cell lines (786‐O and OSRC‐2). To establish a safe and effective AIM2 gene delivery system, we formed the nanoparticles consisting of a folate grafted PEI600‐CyD (H1) nanoparticle‐mediated AIM2 gene (H1/pAIM2) as an effective delivery agent. Delivery of H1/pAIM2 in renal carcinoma cells could remarkably increase the expression of AIM2, and subsequently decrease cell proliferation, migration, and invasion as well as enhance cell apoptosis. In order to evaluate the therapeutic efficacy of AIM2 in vivo, H1/pAIM2 nanoparticles were injected intratumorally into 786‐O‐xenograft mice. Administration of H1/pAIM2 nanoparticles could inhibit the tumour growth as evidenced by reduced tumour volume and weight. Furthermore, Blockade of inflammasome activation triggered by H1/pAIM2 nanoparticles using inflammasome inhibitor YVAD‐CMK abrogated the anti‐tumoral activities of H1/AIM2. These results indicated the therapeutic effect of H1/pAIM2 nanoparticles was mainly attributable to its capability to enhance the inflammasome activation. H1/AIM2 nanoparticles might act as an efficient therapeutic approach for RCC treatment.

## 1 INTRODUCTION

Renal cell carcinoma (RCC) is generally thought to be the most common type of urinary system tumour, accounts for about 3% of all the mortality human malignant tumours, and its incidence is growing by 2% per year.^1^, ^2^ At the beginning of diagnosis or after primary tumour resection, distal metastases already have been found in the majority Of RCC patients.^3^ In recent years, some progress has been made in the development of RCC treatment, but the effective and safe approaches to treat RCC are still not satisfied.^4^, ^5^, ^6^ Therefore, the development of novel therapeutic strategies is urgently needed for RCC treatment.

Absent in melanoma 2 (AIM2) as a cytoplasmic DNA senor, is a family of IFN‐inducible PYHIN (pyrin and HIN200 domain‐containing) proteins.^7^ AIM2 binding to double‐stranded DNA (dsDNA) can recruit the adaptor protein apoptosis‐associated speck‐like protein containing a caspase‐recruitment domain (CARD) (ASC) through homotypic pyrin domain (PYD) interactions. The CARD of ASC binds the CARD of pro‐caspase‐1 to lead to caspase‐1 activation and the production of interleukin‐1β (IL‐1β) and IL‐18 through cleavaging the pro‐IL‐1β and pro‐IL‐18.^8^ The activation of AIM2 inflammasome regulating inflammatory response is well explored in innate immune cells.^7^, ^8^, ^9^, ^10^ Currently, AIM2 as a tumour suppressor is remarkably interest in cancer. The absence of AIM2 in melanoma promoted the cell migration, invasion and disease progression.^11^ The restricted proliferation of cancer cells by over‐expressing AIM2 was found in colon cancer,^12^, ^13^, ^14^, ^15^ breast cancer^16^, ^17^ and prostate cancer.^18^ Thus, these results suggest that AIM2 plays an important role in the development and progression of cancer with decrease or deficiency of AIM2. However, AIM2 serving as a therapeutic target for RCC treatment remains largely unknown.

The efficient gene therapy depends on the ability to efficiently deliver the appropriate therapeutic materials into the target tissues or cells.^19^ Currently, viral vectors including retroviruses, adenoviruses and lentiviruses as delivery system have been widely used in gene therapy.^20^, ^21^, ^22^ However, viral vectors have limited their clinical applications for safety concern such as oncogenic potentials, immuno‐recognition of the viral capsid proteins and generation of various immune responses in vivo.^23^, ^24^ Therefore, the safe and efficient gene carriers are the key to the clinical success of gene therapy. Folate‐grafted PEI600‐CyD (H1) as a non‐viral gene delivery vehicle can effectively condense plasmid DNA to form stable functionalized nanoparticles.^25^ Recent studies have shown a significant success using H1 to deliver genes in vivo.^26^ More importantly, H1‐based delivery system leads to neither elevate enzymes in serum nor a non‐specific immune response in mice, suggesting its low toxicity.^25^, ^26^ Therefore, H1‐based plasmid AIM2 (pAIM2) nanoparticles may be a therapeutic strategy for RCC treatment.

In this study, we observed that AIM2 expression was significantly decreased in RCC specimen and renal carcinoma cell lines, indicated that AIM2 might be served as a therapeutic target for RCC treatment. H1/pAIM2 nanoparticles could remarkably increase the expression of AIM2, and subsequently decrease cell proliferation, enhanced cell apoptosis and inhibit cell migration and invasion in vitro. To evaluate the therapeutic potential of AIM2 in vivo, H1/pAIM2 nanoparticles were intratumorally administrated to 786‐O‐xenograft mice at the early of tumour growth. Our results indicated that administration of H1/pAIM2 could significantly ameliorate the tumour growth of renal carcinoma. The therapeutic efficacy of H1/pAIM2 was mainly attributable to its capability to inhibit the inflammasome activation. Our findings indicated that H1/pAIM2 nanoparticles might represent a novel therapeutic approach for RCC treatment and provide a better understanding of the underlying mechanism of RCC pathogenesis.

## 2 MATERIALS AND METHODS

### 2.1 Human specimens and ethics statement

The tissue specimens of 298 RCC patients who accepted curative surgery without prior treatment at the Department of Pathology of the Affiliated Hospital of Xuzhou Medical University from 2005 to 2008 were enrolled in this study. Twenty cases of normal renal tissues were as the control. The tissue specimens were constructed into tissue microarray (TMA). The TMAs were constructed at the National Engineering Center for Biochip (Shanghai, China) by a contract service and consisted of 298 surgical cases and 20 normal cases. The diameter of every array dot was 1.5 mm, and each dot represented a tissue from one individual specimen.

This study was performed under a protocol approved by the Institutional Review Boards of the Affiliated Hospital of Xuzhou Medical University. All examinations involving human subjects were performed in accordance with relevant guidelines and regulations of the Affiliated Hospital of Xuzhou Medical University after obtaining with informed patient consent. All animal experimental protocols were approved by the guidelines of the Laboratory Animal Ethical Committee of Xuzhou Medical University.

### 2.2 Immunohistochemistry assay

Tissue slides were de‐paraffinized and rehydrated sections were made by epitope retrieval via a heat‐induced protocol. The sections were blocked with 10% BSA and incubated with human AIM2 antibody (1:100 dilution; Santa Cruz Biotechnology), secondary antibody, and then with streptavidin‐peroxidase (Zhongshan Biotech, Beijing, China). The samples were developed by treatment with DAB detection kit (Zhongshan Biotech, Beijing, China) and with hematoxylin to counter stain the nuclei. Immunostaining evaluation was performed as described before.^27^

### 2.3 Formation of H1/pAIM2 nanoparticles

H1 was synthesized according to our previously described method.^25^ Briefly, Folic acid (0.053 g, 0.12 mmol) in DMSO was activated by CDI (0.02 g, 0.12 mmol) using Et3N as the catalyst under nitrogen atmosphere. The reaction mixture was added to the DMSO solution of PEI600‐CyD (0.12 g) synthesized by a method reported previously, and stirred for 24 hours to obtain the crude product of H1, which was purified by dialysis in water for 3 days with dialysis tubing (MWCO, 12 kD). Pale yellow powder of H1 was obtained after lyophilization for 2 days.

Human AIM2 full length from plasmid pEFBOS‐AIM2 was subcloned into pcDNA3.1 using EcoRI and BamHI. pcDNA3.1‐AIM2 (pAIM2) was transformed into Escherichia coli (DH5a) competent cells, propagated in LB broth supplemented with 100 mg/mL Ampicillin and purified from DH5a growing overnight using Qiagen EndoFree Plasmid Mega kit.

To prepare H1/pAIM2 nanoparticle polyplexes, H1 and plasmid were mixed and dissolved in 5% glucose solution at an N/P ratio of 20:1 in the same volume. The nanoparticle polyplexes were stabilized for 10 minutes at the room temperature prior to tranfection or injection.

### 2.4 H1/pAIM2 transfection in vitro

The human RCC cell lines 786‐O and OSRC‐2 (ATCC) cells were cultured in RPMI 1640 medium (Gibco, Invitrogen) supplemented with 10% fetal bovine serum (Gibco, Invitrogen). These two cell lines were both incubated in 5% CO_2_ atmosphere at 37°C. For cell transfection assays, 5 × 10^5^ cells at about 70%‐80% confluence were incubated with 4 μg H1 packaged pAIM2 in 35‐mm dishes. Same amount of H1/pcDNA3.1 vector was used as a control.

### 2.5 Western bloting

Total samples lysates containing approximately equal amounts of proteins were solubilized in sodium dodecyl sulfate (SDS)‐sample buffer by heating at 95°C for 10 minutes, separated by 12% SDS‐polyacrylamide gel electrophoresis, transferred to polyvinylidene difluoride (PVDF) membranes and incubated with primary antibodies against AIM2, ASC (Santa Cruz Biotechnology), pro‐Caspase‐1, Caspase‐1, Pro‐IL‐1β, IL‐1β, GAPDH (Cell Signaling Technology) overnight at 4°C. After washing with Tris‐buffered saline containing 0.1% tween‐20 (TBST), the membranes were incubated with horseradish peroxidase (HRP)‐linked secondary antibody (Southern Biotech, Birmingham, AL, USA) for 2 hours at room temperature and rinsed with TBST. Then, the immunoreactive bands were detected by chemiluminescence using the ECL (Thermo Fisher Scientific).

### 2.6 Cell proliferation assay

The treated cells were plated into 96‐well plates at a density of 3 × 10^3^ cells/well, respectively, and cells were incubated 37°C for 24 hours, 48 hours and 72 hours. Ten microlitres solution of Cell Counting Kit‐8 (CCK‐8, Beyotime, Nantong, China) were added to each well and incubated at 37°C for 1 hour. Absorbance at 450 nm was measured on an ELX‐800 spectrometer reader (Bio‐Tek Instruments).

### 2.7 Flow cytometry analysis

The collected cells were washed twice with PBS, and then fixed with fixation buffer (BD Biosciences) for 30 minutes at 4°C. The fixed cells were permeabilized with permeabilization solution (BD Biosciences) at room temperature for 0.5 hours.

Cells were incubated with anti‐human AIM2 antibody (diluted 1:200; Santa Cruz) in PBS containing 2% FBS for overnight at 4°C. Isotype‐matched anti‐human IgG served as control. FITC conjugated IgG (diluted 1:100; Biolegend) was used as secondary antibody. Then labelled cells were analysed using a BD FACSCanto II flow cytometer with FACSDiva software (BD Biosciences).

### 2.8 Colony formation assay

For the colony formation assay, cells were transfected with pAIM2 or Vector, as described above. Twenty four hours later, transfected cells were trypsinized, counted and seeded in 6‐well plate at a density of 1 × 10^3^ cells per well. After 7 days of incubation, survival colonies were fixed with 4% paraformaldehyde for 10 minutes and stained with 0.5% of crystal violet and counted under the microscope.

### 2.9 Cell apoptosis detection

Cells were trypsinized and rinsed twice with PBS at 72 hours post‐treatment. Flow cytometry analysis of cell apoptosis was performed using an Annexin V‐FITC/PI apoptosis detection kit (BD Biosciences) according to the manufacturer's instructions.

### 2.10 Cell migration and invasion assay

Cell migration and invasion assays were performed using modified 2‐chamber plates with a pore size of 8 μm. For migration assay, 5 × 10^4^ cells suspended in 200 μL of serum‐free medium were seeded on the upper compartment of 24‐well transwell culture chamber, and 600 μL of complete medium was added to the lower compartment. After 12‐hour incubation at 37°C, cells were fixed with methanol. For invasion assay, 30 μL of 1:8 diluted Matrigel (BD Biosciences) in serum‐free medium was added to the upper compartment of 24‐well transwell culture chamber. On the upper compartment, 6 × 10^4^ cells suspended in 200 μL of serum‐free medium were seeded and 600 μL of complete medium was added to the lower compartment. After 24‐hour incubation at 37°C, cells were fixed with methanol. Migrated or invaded cells on the lower side of the filter were stained with crystal violet and counted.

### 2.11 Wound healing assay

Cells treated with H1/pAIM2 or control were grown to confluency, a wound line was made by scraping a closed Pasteur pipette tip across the confluent cell layer. Then, cells were washed three times with PBS to remove detached cells and debris. The size of the wound was observed and measured after 24 hours.

### 2.12 Caspase‐1 activity assay

The caspase‐1 activity was detected by the colorimetric caspase‐1 assay kits (Beyotime Biotechnology). Briefly, the extracts were centrifuged at 12 000 g for 10 minutes, and the supernatant was collected. A volume of supernatant equivalent to 100 μg of protein were incubated with 50 mmol/L substrate (Ac‐YVAD‐MCA for caspase‐1 proteinase), 0.05 mol/L NaCl, 2.5 mmol/L dithiothreitol and 10 mmol/L HEPES, pH 7.5, in 300 μL of the reaction mixture for 1 hour at 37°C. The fluorescence intensity of the released AMC was measured with a spectrofluorometer at 380 nm/460 nm excitation/emission wavelength. One unit was defined as the amount of the enzyme required to release 1 μmol AMC per hour at 37°C.

### 2.13 ELISA measurement of cytokines

To assess protein levels of IL‐1β in culture supernatant or in homogenized tumour tissues and IL‐6 in serum of mice, enzyme‐linked immunosorbent assays (ELISA) were performed according to the manufacturer's instructions (eBioscience).

### 2.14 Animal model

Nude mice (BALB/c‐nu/nu) aged 4‐6 weeks were purchased from Vital River Laboratory Animal Technology Co., Ltd (Beijing, China) and housed under specific pathogen‐free (SPF) conditions. To establish subcutaneous human RCC xenograft mouse model, 786‐O cells (2 × 10^6^ cells) growing in the logarithmic phase were subcutaneously injected into the hind legs of nude mice. On day 7 post‐injection when the tumour reached about 50 mm^3^ in volume, the mice were randomly divided into four groups (n = 8 per group), namely control, control+inhibitor, H1/pAIM2 and H1/pAIM2 + inhibitor groups. On the day of randomization (day 0), H1/pAIM2 nanoparticles (50 μg/mouse) or control were administered by intratumoral injection weekly over the next 3 weeks. Meanwhile, the inflammasome inhibitor AC‐YVAD‐CMK (10 mg/kg, Sigma‐Aldrich) was administrated intraperitoneally in treated‐mice. The survival status of tumour‐bearing mice was monitored daily. The body weight and tumour diameter were measured every 3 days. Tumour volume was calculated using the following equation: Volume = (*A* × *B* ^2^/2) (*A* refers to the long diameter and *B* refers to the short diameter). Animals were killed 2 weeks later, tumour tissues were surgically excised from the mice and tumour weight was evaluated.

### 2.15 Pathological analysis

For routine histological analysis, tissues were surgically resected and fixed in 4% paraformaldehyde (Sigma‐Aldrich), embedded in paraffin and cut into sections 4 μm sections. H&E staining was performed according to the manufacturer's instructions, and the sections were assessed by a pathologist blinded to treatment group. Pictures were acquired with Nikon SCLIPSS TE2000‐S microscope (Nikon) equipped with ACT‐1 software. Original magnification was ×100.

### 2.16 Statistical analysis

Statistical analysis was performed with SPSS software (version 16.0, Armonk, NY, USA) and expressed as means ± SD. Statistical significance was evaluated using two‐tailed Student's *t* test. Multiple comparisons were performed using one‐way ANOVA. The statistical significance level was set as *P < 0.05; **P < 0.01; ***P < 0.001.

## 3 RESULTS

### 3.1 AIM2 expression is significantly increased in RCC patient tissues and renal cancer 786‐O or OSRC‐2 cell lines

**Figure 1:**
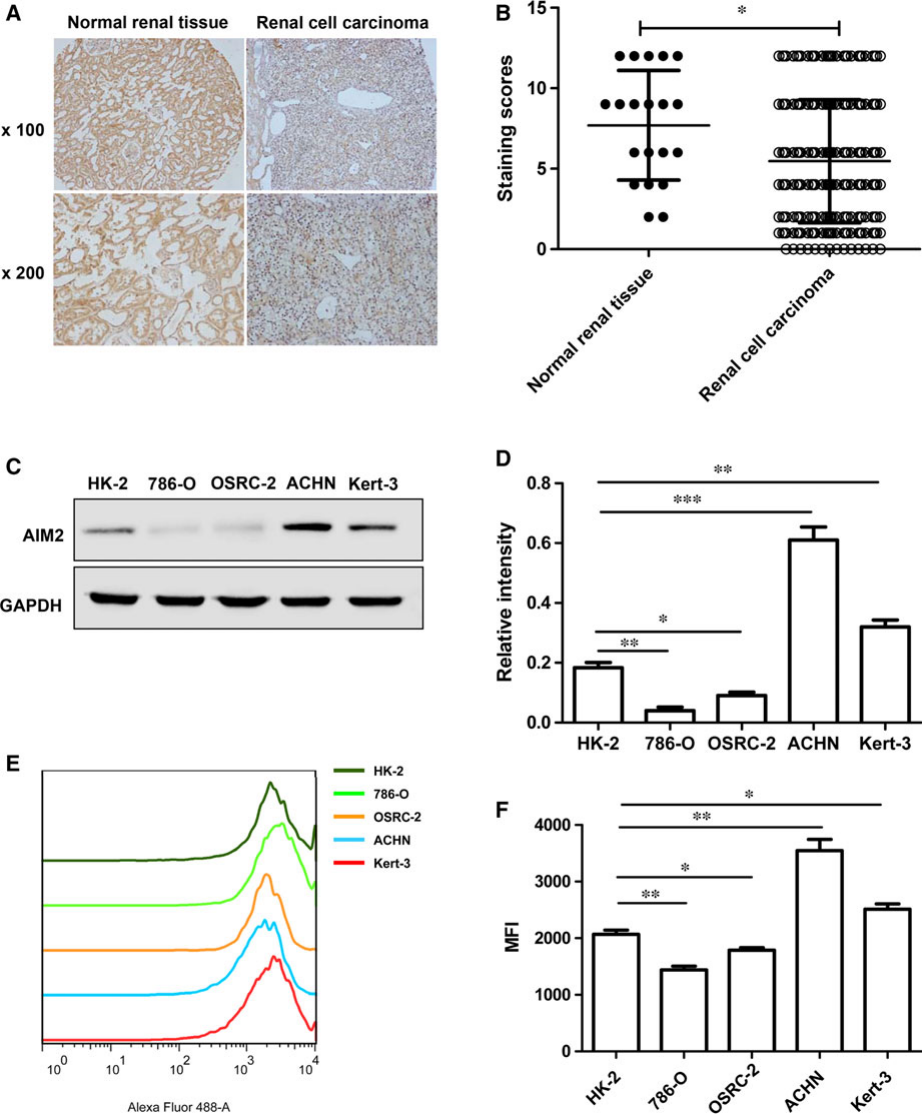
AIM2 expression was decreased in human renal cell carcinoma (RCC) and renal cell lines. (A). This represents immunohistochemical staining of AIM2 in RCC and normal renal tissues, top panel,×100, bottom panel, ×200. (B). Staining scores of AIM2 were evaluated in (A), the immunohistochemical staining data were available from 20 normal renal tissues and 298 RCC. (C). AIM2 expression was evaluated in both 786‐O and OSRC‐2 cell lines by Western blot. (D). The relative values were estimated in the band intensity of each band normalized by GAPDH. (E). The expression levels of AIM2 were detected by flowcytometry in HK‐2, 786‐O, OSCR‐2, ACHN and Kert‐3 cells. (F). The data shown as statistical analysis of the mean fluorescence intensity in (E). Data represent the means of three independent experiments. Data are shown as means ± SD. The different significance was set at *P < 0.05, **P < 0.01 and ***P < 0.001

To determine whether AIM2 was involved in pathogenesis of RCC, we firstly detected the expression of AIM2 in 298 specimens of RCC patients. As shown in Figure 1A and B, Immunohistochemical staining and staining scores showed that AIM2 expression was lower in RCC tissues than normal renal tissues. Furthermore, we also detected the local expression of AIM2 in renal cancer cells. Compared with normal renal HK‐2 cells, Western blot analysis showed that the local levels of AIM2 were reduced in 786‐O or OSRC‐2 cells, while increased in ACHN or Kert‐3 cells (Figure 1C and D). Absent in melanoma 2 local levels were further confirmed by flow cytometry in these cell lines. Consistently, the low MFI of AIM2 was observed in 786‐O and OSRC‐2 cells and the high MFI of AIM2 was seen in ACHN and Kert‐3 as compared with HK‐2 cells (Figure 1E and F). These results indicated that the decreased AIM2 expression might be involved in pathogenesis of RCC, and the increase of AIM2 expression might serve a therapeutic strategy for RCC treatment.

### 3.2 H1/AIM2 inhibited renal cancer cell proliferation and promoted cell apoptosis

**Figure 2:**
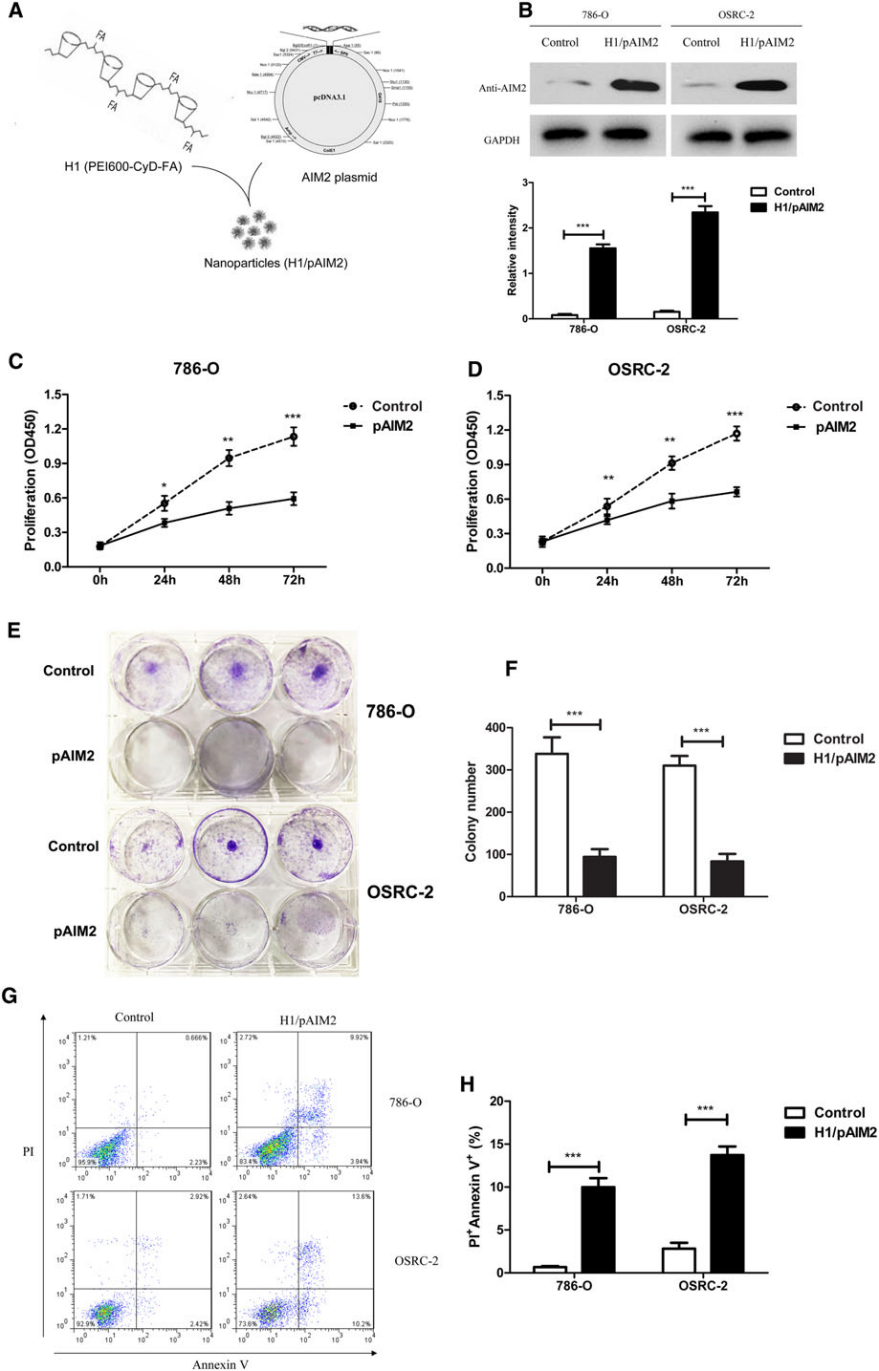
H1/AIM2 nanoparticles inhibited cell proliferation and promoted cell apoptosis in renal cancer cells. (A). A schematic of H1/pAIM2 nanoparticle formation. (B). Forty‐eight hours after H1/pAIM2 transfection, AIM2 expression was evaluated by Western blot in both 786‐O and OSRC‐2 cell lines. (B). The values of the band intensity below the figure represent the densitometric estimation of each band normalized by GAPDH. (C and D). CCK‐8 cell proliferation assay was performed after H1/AIM2‐treated in 786‐O and OSRC‐2. (E and F). The colony formation capability of H1/pAIM2 or control‐treated renal cancer cells were detected at day 7. (G) H1/pAIM2 The cell apoptosis was detected by flow cytometry in 786‐O and OSRC‐2. (H). Statistical histograms of the percentage of apoptotic cells among H1/pAIM2‐, and control‐treated 786‐O and OSRC‐2 cells, respectively. All experiments were carried out in triplicate. Data are shown as means ± SD. The different significance was set at *P < 0.05, **P < 0.01 and ***P < 0.001

Our previous study has demonstrated that H1 nanoparticles were an effective delivery system for gene expression in vitro and in vivo.^26^ Thus, H1/pAIM2 or control nanoparticles were prepared (Figure 2A) and transiently transfected into 786‐O or OSRC‐2 cells. The result showed AIM2 expression was significantly increased in both renal cancer cell lines as compared with control (Figure 2B). Then, we further investigated the function of AIM2 in the proliferation of renal cancer cells. In the CCK‐8 cell proliferation assay, H1/AIM2 group showed a significantly inhibited effect on cell proliferation both in 786‐O and OSRC‐2 cells (Figure 2C and D). Moreover, the colony formation assay showed that H1/AIM2 significantly decreased capabilities of colony formation in renal cancer cells compared with control (Figure 2E and F). The apoptosis of renal cancer cells was also increased in H1/pAIM2 group in contrast to control group (Figure 2G and H). These results indicated that H1/AIM2 could inhibit renal cancer cell proliferation and enhance cell apoptosis.

### 3.3 H1/AIM2 suppressed renal cancer cells migration and invasion in vitro

**Figure 3:**
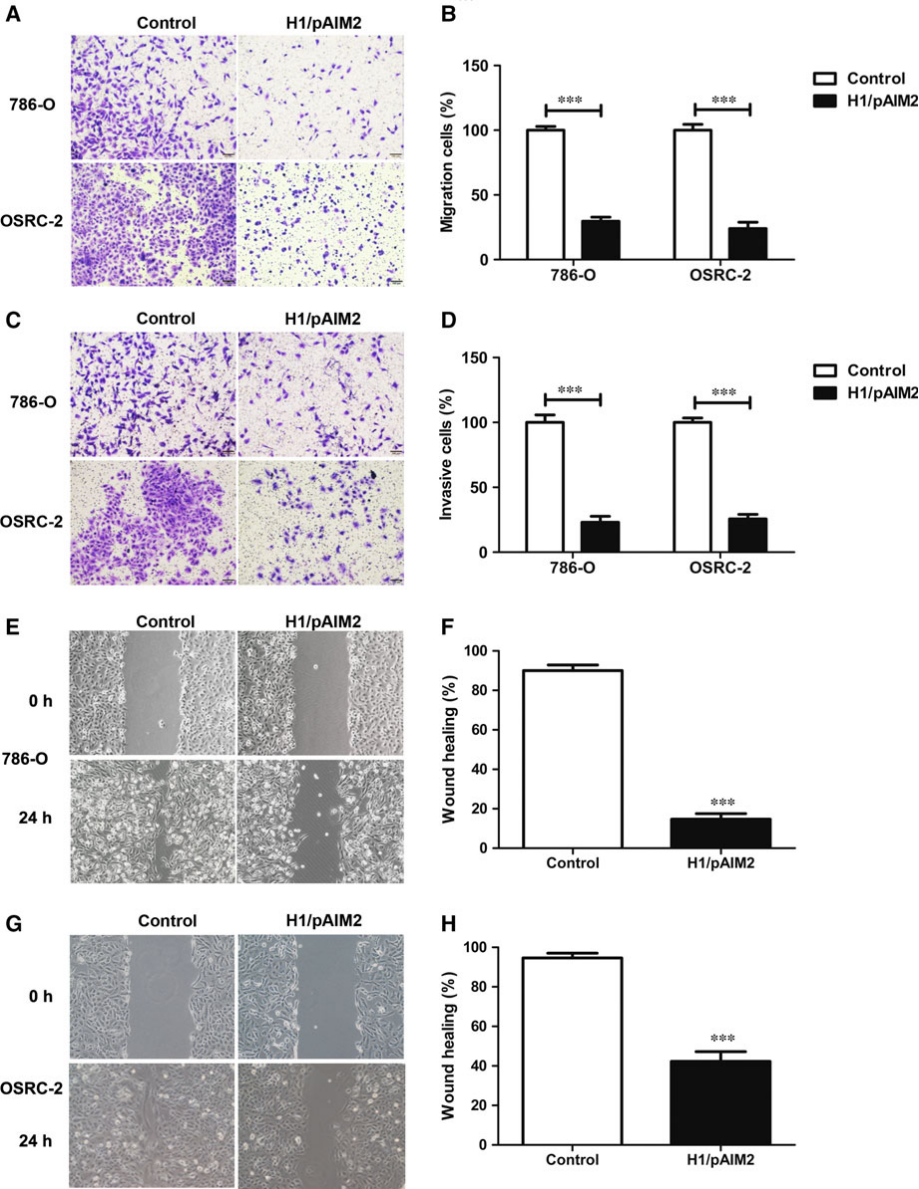
H1/pAIM2 nanoparticles suppressed migration and invasion of renal cancer cells. (A and B). The migration of 786‐O and OSRC‐2 cells was detected in H1/pAIM2 group or control group. (C and D). The invasion of 786‐O and OSRC‐2 cells was detected. (E and F). The wound healing of 786‐O cell line was measured. (G and H). The wound healing of OSRC‐2 cell line was measured. Data are from one representative experiment of three performed and presented as the mean ± SD. The different significance was set at ***P < 0.001

We next investigated the role of H1/AIM2 in migration and invasion of renal cancer cells. The results of cell migration assay showed that the cell migration and percentage in 786‐O or OSRC‐2 cells were obviously reduced in H1/pAIM2 group (Figure 3A and B). Consistently, H1/pAIM2 suppressed the cell invasive ability, which was confirmed by the reduced percentage of invasive renal cancer cells (Figure 3C and D). The effect of AIM2 on the migration of renal cancer cells was further examined by wound healing assay. H1/pAIM2 group showed the slower speed of wound healing in 786‐O or OSRC‐2 cells by detecting the width of the scratches than control group (Figure 3E‐H). These results indicated that H1/AIM2 could inhibit the migration and invasion of renal cancer cells.

### 3.4 Administration of H1/AIM2 reduced the tumour growth in renal cancer cell‐xenografted nude mice

**Figure 4:**
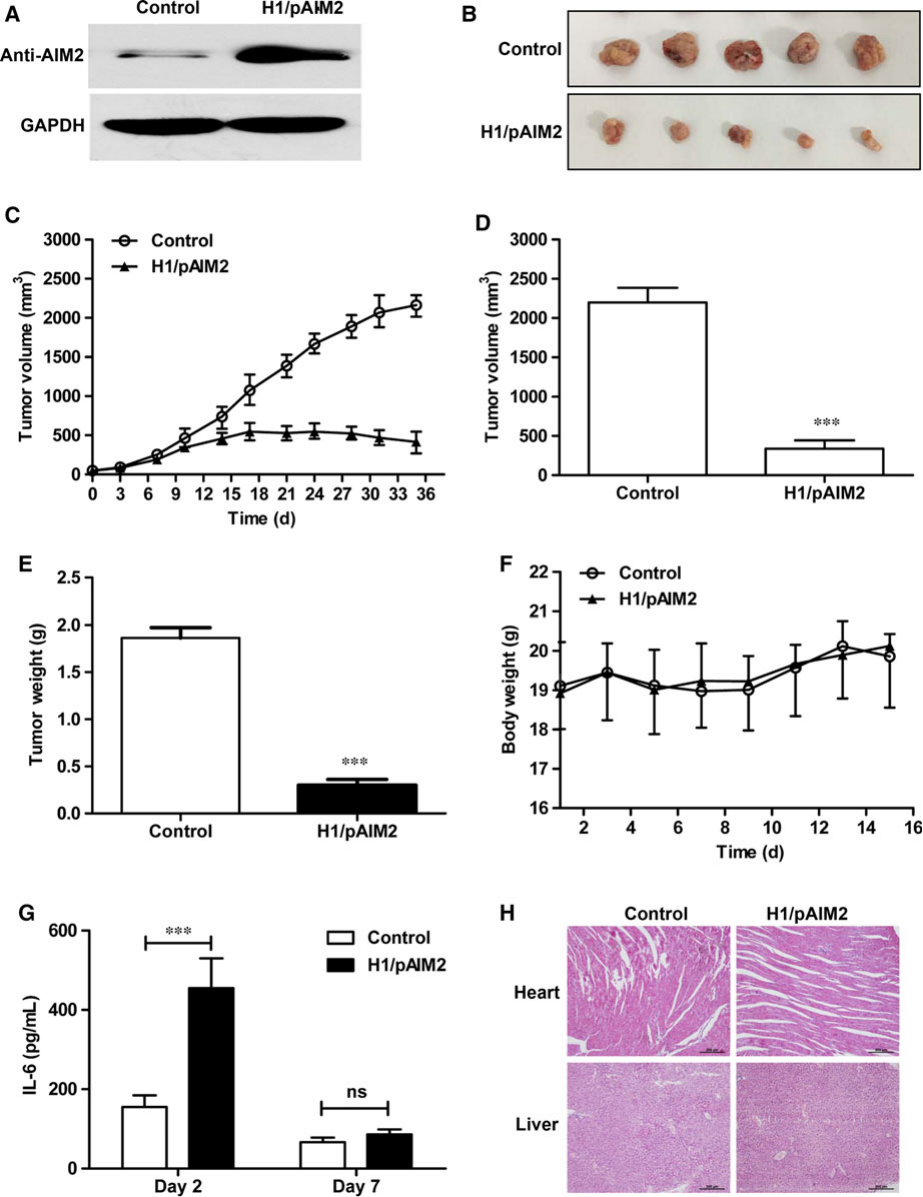
Administration of H11/AIM2 nanoparticles inhibits the tumour growth of 786‐O cell‐xenografted nude mice. The 786‐O cell‐xenografted nude mice were administrated intratumorally H1/pAIM2 or control (50 μg/mouse) on day 7 post‐injection when the tumour reached about 50 mm^3^ in volume weekly for total 3 times. (A). AIM2 expression was detected by western blot in tumour tissues. (B). The present images of tumour excised from mice treated with H1/pAIM2 or control after 35 d of growth in vivo. (C). Tumour progression of 786‐O cell‐xenograft was evaluated by measurement of tumour volume twice per 7 d from day 0 to day 35. (D). The tumour volumes were measured at day 35 after tumour inoculation. (E). The tumour weights were monitored at day 35 after tumour inoculation. (F). Body weights of mice were detected per 2 d from day 1 to day 35. (G). At day 2 or 7 after the initial administration, the serum were collected, the levels of IL‐6 were determined by ELISA assay. (H) At day 35 after tumour inoculation, the pathology was evaluated by H&E staining of heart or liver tissues. Each experiment was performed independently at least three times and the results of one representative experiment are shown. Data are means ± SD, ***P < 0.001, ns, no significant difference

To further evaluate the therapeutic efficacy of H1/AIM2 in RCC, the subcutaneous 786‐O‐xenograft model established in nude mice were intratumorally administrated with H1/pAIM2 or control. We firstly detected the expression efficiency of H1/AIM2 in tumour, and found that the higher AIM2 expression was observed in H1/pAIM2 group than control group (Figure 4A). At day 35 after tumour inoculation, the significantly suppressed tumour growth was observed in H1/AIM2‐treated group compared with control‐treated group (Figure 4B and C). Accordingly, the volume and weight of tumour were decreased in H1/AIM2‐treated group (Figure 4D and E). Moreover, H1/pAIM2 could not significantly affect the body weight (Figure 4F) and elevate the level of cytokines, like IIL‐6 in serum (Figure 4G). There is a non‐specific immune response evidenced by normal pathological tissue of heart and liver in H1/pAIM2‐treated mice (Figure 4H), indicated suggesting its low toxicity. Taken together, these data indicated that H1/AIM2 treatment with low toxicity could effectively prevent the aggravation of tumour.

### 3.5 H1/AIM2 enhanced the inflammasome activation of AIM2 in renal cancer cells

**Figure 5:**
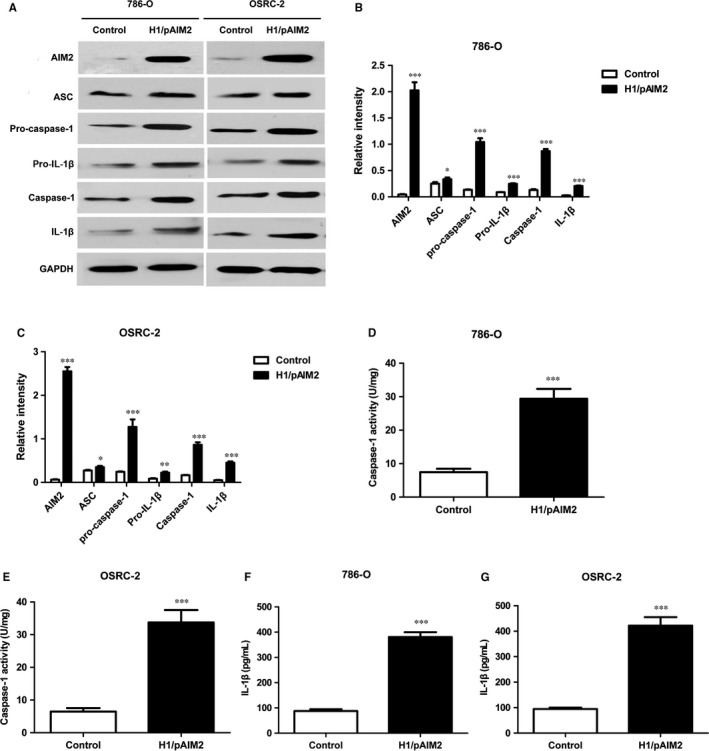
H1/pAIM2 enhanced the activation of inflammasome in renal cancer cells. (A). The levels of AIM2, ASC, Pro‐caspase‐1, Pro‐IL‐β, Caspase‐1, IL‐1β were measured by Western blot in H1/AIM2 or control‐treated 786‐O or OSRC‐2 cell lines. (B and C). The statistical analysis of the band intensity represents the densitometric estimation of each band normalized by GAPDH in (A). (D and E). The levels of caspase‐1 were measured in H1/AIM2‐ or control‐treated 786‐O or OSRC‐2 cell lines. (F and G). The levels of IL‐1β were measured in treated renal cancer cell lines. Data are from one representative experiment of three performed and presented as the mean ± SD. The different significance was set at *P < 0.05, **P < 0.01 and ***P < 0.001

The inflammasome is a multiprotein complex that has recently been shown to the corresponding contribution of innate immunity pathways in suppressing tumour growth.^15^, ^28^ Thus, we are attracted to define whether the anti‐tumour effect of H1/AIM2 in RCC is mediated by AIM2 inflammasome. Our data indicated that the increased inflammasome components (AIM2/casepase‐1/IL‐1β) were observed in H1/pAIM2‐treated 786‐O or OSRC‐2 cells compared the control‐treated cells (Figure 5A‐C). Equally, caspase‐1 activity and IL‐1β production were significantly up‐regulated in these H1/pAIM2‐treated cells (Figure 5D‐G), indicated that AIM2 inflammasome was formed and activated. These results indicated that H1/AIM2 exerting its anti‐tumour effect was associated with AIM2 inflammasome activation.

### 3.6 H1/AIM2 inhibited the cell migration and invasion and exerted its anti‐tumour effect through AIM2 inflammasome activation

**Figure 6:**
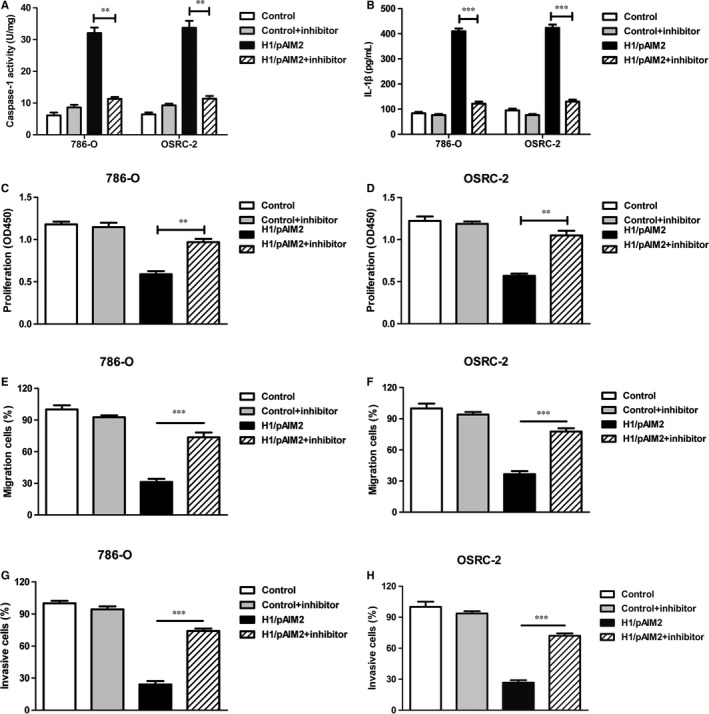
H1/AIM2 inhibits the proliferation, migration and invasion of renal cancer cells through enhanced the inflammasome activation. 786‐O and AOSRC‐2 cells in 35‐mm dish were treated by 4 μg H1/pAIM2 or control with or without Ac‐YVAD‐MCA (50 μmol/L). (A). Forty‐eight hours after treatment, the levels of caspase‐1 were measured in 786‐O and OSRC‐2. (B). ELISA analysis of IL‐1β in renal cancer cells. (C and D). CCK‐8 cell proliferation assay was performed in renal cancer cells. (E and F). The migration was detected in renal cancer cells. (G and H). The invasion was detected in renal cancer cells. Data are from one representative experiment of three performed and presented as the mean ± SD. The different significance was set at **P < 0.01, and ***P < 0.001

**Figure 7:**
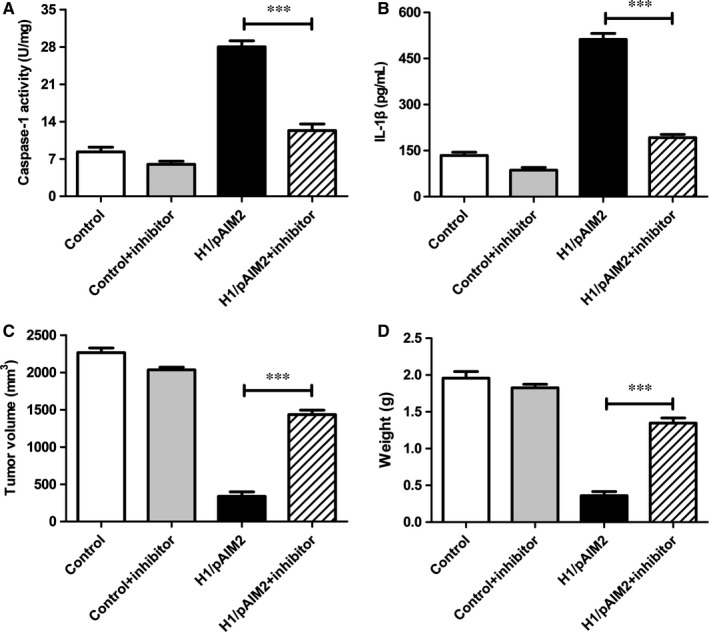
The inhibitor Ac‐YVAD‐MCA abrogated the anti‐tumour function of H1/pAIM2 in 786‐O cell‐xenograft model. The inhibitor Ac‐YVAD‐MCA was administrated (10 mg/kg) intraperitoneally in 786‐O cell‐xenografted nude mice treated with H1/pAIM2 or control. (A). At day 35 after tumour inoculation, the levels of caspase‐1 were measured in tumour tissues. (B). The levels of IL‐1β were measured in tumour tissues. (C). The tumour xenografts were excised at day 35 after tumour inoculation, the tumour volumes were measured. (D). The tumour weights were measured. Data are from one representative experiment of three performed and presented as the mean ± SD. The different significance was set at ***P < 0.001

To further validate our hypothesis that H1/AIM2 could suppress the migration and invasion of RCC by enhancing the inflammasome activation, renal cancer cells was treated with H1/pAIM2 nanoparticles followed with or without the downstream inflammatory caspase inhibitors YVAD‐CMK. The results showed that the inflammasome activation was blocked in 786‐O or OSRC‐2 cells (Figure 6A and B) and the inhibited ability of cell proliferation, migration and invasion was prevented in Yvad‐CMK‐treated H1/pAIM2 group (Figure 6C‐H). Further study showed that after blocking the effect of inflammasome activation by administration of YVAD‐CMK in vivo, the levels of capase‐1 and IL‐1β remarkably were reduced in tumour (Figure 7A and B). Furthermore, the rescued malignant behaviours including tumour volume (Figure 7C) and tumour weight (Figure 7D) were observed in YVAD‐CMK‐treated H1/pAIM2 group compared with H1/pAIM2 group. These data indicated that the therapeutic efficacy of anti‐tumour by H1/pAIM2 is depended on the inflammasome activation.

## 4 DISCUSSION

Renal cell carcinoma has a worse prognosis and easily develop metastatic disease, which are usually incurable, and the median survival of metastatic RCC patients is significantly worse.^29^ Currently, the treatments for RCC have evolved from conventional drugs to biologic targeting therapies, among which cytokines are the most crucial therapeutic targets.^30^, ^31^ However, the existing therapies for RCC are not satisfactory owing to the low efficacy in suppressing disease activity as well as the adverse effects in patients. In this study, H1 nanoparticles with a low toxicity were applied in the delivery system of targeting therapeutic.

Absent in melanoma 2 as DNA sensor, a range of cellular defence mechanisms including cytokines secretion are triggered to activate the immune system.^32^ Moreover, the down‐regulated AIM2 expression is a risk factor in tumour progression.^14^, ^16^ Thus, AIM2 plays an important dual role in both innate immunity and tumour pathology. Absent in melanoma 2 as a therapeutic target for RCC treatment is not fully clarified in current study. In this study, the therapeutic effect of AIM2 packaged with H1 nanoparticles was evaluated in RCC. Our results demonstrated that administration of H1/pAIM2 nanoparticles could alleviate the disease severity in 786‐O cell‐xenografted nude mice. The therapeutic effect of H1/pAIM2 is mainly attributed to its ability to inhibit the activation of inflammasome. Therefore, we believed that gene therapy by H1/AIM2 nanoparticles could be an alternative solution to alleviate the tumour growth through enhancing the inflammasome and proinflammatory cytokines.

Absent in melanoma 2 function was not clearly understood in RCC. Therefore, we used TMA technology and IHC to investigate the expression of AIM2 in RCC. The results present evidence that AIM2 expression was decreased in RCC tissues compared with normal cancer tissues. Meanwhile, we detected the expression of AIM2 in renal cancer cell lines. Compared with HK‐2 cells, the reduced expression of AIM2 was found in 786‐O and OSRC‐2 cells, but the increased expression of AIM2 was observed in ACHN and Kert‐3 cells. These results implied that AIM2 might have versatile biological activities in different cancer cells. It is well known that tumour cell proliferation, migration and invasion are main processes of metastasis, which remains the major cause of mortality in patients with RCC. We suspected that over‐expression of AIM2 by H1/pAIM2 might inhibit RCC proliferation, migration and invasion. Our data demonstrated that H1/AIM2 could inhibit cell proliferation, migration and invasion, indicated that AIM2 does regulate the metastasis of RCC.

The inflammasome responds to multiple danger signals, including a decrease in cytosolic potassium concentrations and an increase in cytosolic DNA levels, dying tumour cells and bacterial products.^33^ H1/AIM2 may contribute to the stimulation of the inflammasome activation by recognizing dsDNA from dying tumour cells. Previous study revealed that AIM2 inflammasome in HCC cells suppressed mammalian target of rapamycin (mTOR)‐S6K1 pathway,^28^ and suggested that mTOR pathway might be involved in AIM2‐induced downstream pathway. The activation of inflammasome has been reported to inhibit cancer cell invasion and metastasis, angiogenesis or tumorigenesis in model systems, suggesting that the defective of inflammasome in tumour cells is a tumour suppression mechanism.^34^ Our data demonstrated that H1/pAIM2 expression significantly enhanced the expression of inflammasome competent (AIM2/Caspase‐1/IL‐1β) in renal cancer cell lines. Accordingly, the inhibition of cell invasion and metastasis by H1/pAIM2 was blocked by inflammasome inhibitor YVAD‐CMK in vitro. In vivo experiment, we observed the prevent effect of the reduced growth tumour in YVAD‐CMK‐treated 786‐O cell‐xenografted nude mice administrated with H1/pAIM2. These data indicated that the therapeutic effect of H1/pAIM2 in the inhibition of cell migration and invasion was associated with enhancing the activation of inflammasome. Taken together, AIM2 might serve as a common danger sensor in different cancer cell types, which helps the host cells to be exempt from immune inflammation, and thus maintains the intracellular homeostasis and sanctity. In the future, we would further investigate the mechanism of AIM2 inflammasome on the side of cancer cell apoptosis.

In this study, our results suggested that H1/AIM2 nanoparticles could inhibit malignancies of renal cancer through enhancing the inflammasome pathway, which suggested an effective treatment of H1/AIM2 against renal cancer. Therefore, it indicated that therapeutic strategy by H1/AIM2 might provide a new way for manipulating renal cancer.

## CONFLICT OF INTERESTS

The authors declare that they have no competing interests.

## AUTHOR CONTRIBUTIONS

Conceived and designed the project: DC, JZ; Performed the project: DC, ZL, NL; Analysed the data: DC; Contributed reagents/materials/analysis tools: DC, GW, HL, NL, JZ; Wrote the paper: DC. All authors read and approved the final manuscript.
